# Supplementary material for: Women with HPV-Driven Anal and Genital Disease: Investigating the Patient Cohort in England
Source: Cancers (Basel). 2025 Dec 12;17(24):3970. doi: 10.3390/cancers17243970 (PMC12731167; doi:10.3390/cancers17243970)
Supplement: Supplementary file 1 [file cancers-17-03970-s001.zip › cancers-3972064-supplementary.pdf]

Table S1. Age at diagnosis for each anogenital pathology.

Age Groups	Cervical Cancer (%)	Cervical HSIL (%)	Vaginal Cancer (%)	Vaginal HSIL (%)	Vulval Cancer (%)	Vulval HSIL (%)	Anal Cancer (%)	Anal HSIL (%)	Total (%)
**<40y**	36 (50)	439 (80.7)	4 (21.1)	32 (61.5)	93 (33.6)	324 (56.5)	164 (22.3)	258 (42.9)	1350 (47)
**40-54y**	14 (19.4)	65 (11.9)	3 (15.8)	7 (13.5)	73 (26.4)	131 (22.9)	225 (30.7)	201 (33.4)	719 (25)
**55-74y**	19 (26.4)	36 (6.6)	9 (47.4)	11 (21.2)	79 (28.5)	104 (18.2)	259 (35.3)	127 (21.1)	644 (22.4)
**75+y**	3 (4.2)	4 (0.7)	3 (15.8)	2 (3.8)	32 (11.6)	14 (2.4)	86 (11.7)	15 (2.5)	159 (5.5)
**Total (%)**	72 (100)	544 (100)	19 (100)	52 (100)	277 (100)	573 (100)	734 (100)	601 (100)	2872 (100)

Table S2. Primary treatment for each anogenital pathology.

Treatment 1	Cervical Cancer (%)	Cervical HSIL (%)	Vaginal Cancer (%)	Vaginal HSIL (%)	Vulval Cancer (%)	Vulval HSIL (%)	Anal Cancer (%)	Anal HSIL (%)	Total (%)
**CRT**	2 (2.8)	0 (0.0)	1 (5.3)	0 (0.0)	6 (2.2)	0 (0.0)	142 (19.3)	0 (0.0)	151 (5.3)
**Cryotherapy**	0 (0.0)	3 (0.6)	0 (0.0)	0 (0.0)	0 (0.0)	0 (0.0)	0 (0.0)	0 (0.0)	3 (0.1)
**Laser Therapy**	0 (0.0)	26 (4.8)	0 (0.0)	4 (7.7)	1 (0.4)	18 (3.1)	0 (0.0)	11 (1.8)	60 (2.1)
**Radiotherapy**	6 (8.3)	0 (0.0)	1 (5.3)	0 (0.0)	2 (0.7)	0 (0.0)	42 (5.7)	1 (0.2)	52 (1.8)
**Surgery**	51 (70.8)	381 (70.0)	13 (6.8)	29 (55.8)	244 (88.1)	457 (79.8)	472 (64.3)	409 (68.1)	2056 (71.6)
**Chemotherapy**	0 (0.0)	0 (0.0)	0 (0.0)	0 (0.0)	1 (0.4)	0 (0.0)	14 (1.9)	0 (0.0)	15 (0.5)
**Brachytherapy**	0 (0.0)	0 (0.0)	1 (5.3)	0 (0.0)	0 (0.0)	0 (0.0)	0 (0.0)	0 (0.0)	1 (0.03)
**Missing or Unknown**	13 (18.1)	134 (18.1)	3 (15.8)	19 (36.5)	23 (8.3)	98 (17.1)	64 (8.7)	180 (30.0)	534 (18.6)
**Total (%)**	72 (100)	544 (100)	19 (100)	52 (100)	277 (100)	573 (100)	734 (100)	601 (100)	2872 (100)

Table S3. Secondary treatment for each anogenital pathology.

Treatment 2	Cervical Cancer (%)	Cervical HSIL (%)	Vaginal Cancer (%)	Vaginal HSIL (%)	Vulval Cancer (%)	Vulval HSIL (%)	Anal Cancer (%)	Anal HSIL (%)	Total (%)
**CRT**	8 (33.3)	0 (0.0)	3 (50.0)	0 (0.0)	14 (20.0)	0 (0.0)	226 (69.5)	0 (0.0)	251 (43.1)
**Cryotherapy**	0 (0.0)	0 (0.0)	0 (0.0)	0 (0.0)	0 (0.0)	0 (0.0)	0 (0.0)	0 (0.0)	0 (0.0)
**Laser Therapy**	0 (0.0)	8 (25.8)	0 (0.0)	2 (50.0)	0 (0.0)	32 (37.6)	0 (0.0)	20 (54.1)	62 (10.7)
**Radiotherapy**	9 (37.5)	0 (0.0)	1 (16.7)	0 (0.0)	20 (28.6)	0 (0.0)	56 (17.2)	0 (0.0)	86 (14.8)
**Surgery**	0 (0.0)	3 (9.7)	0 (0.0)	1 (25.0)	1 (1.4)	6 (7.1)	4 (1.2)	2 (5.4)	17 (2.9)
**Chemotherapy**	1 (4.2)	0 (0.0)	0 (0.0)	0 (0.0)	7 (10.0)	1 (1.2)	9 (2.8)	0 (0.0)	18 (3.1)
**Brachytherapy**	2 (8.3)	0 (0.0)	0 (0.0)	0 (0.0)	0 (0.0)	0 (0.0)	0 (0.0)	0 (0.0)	2 (0.3)
**Missing or Unknown**	4 (16.7)	20 (64.5)	2 (33.3)	1 (25.0)	28 (40.0)	46 (54.1)	30 (9.2)	15 (40.5)	146 (25.1)
**Total (%)**	24 (100)	31 (100)	6 (100)	4 (100)	70 (100)	85 (100)	325 (100)	37 (100)	582 (100)

Table S4. Multivariate logistic regression looking at the relationships between ethnicity and anogenital cancer staging as well as deprivation and anogenital cancer staging.

Late vs Early Stage		
**Ethnicity**	**[1]OR [95% [2]CI]**	**P-value**
White (Ref.)	-	-
Asian	0.05 [0.00, 84632.44]	0.68
Black	1.37 [0.43, 4.36]	0.59
Mixed & Other	Not interpretable	Not interpretable
Unknown	0.41 [0.08, 2.02]	0.28
**Deprivation**	**[1]OR [95% [2]CI]**	**P-value**
High Deprivation (Ref.)	-	-
Low Deprivation	0.78 [0.50, 1.22]	0.27
**HSIL vs Early Stage**		
**Ethnicity**	**[1]OR [95% [2]CI]**	**P-value**
White (Ref.)	-	-
Asian	Not interpretable	Not interpretable
Black	1.59 [0.68, 3.73]	0.29
Mixed & Other	0.29 [0.07, 1.18]	0.08
Unknown	0.62 [0.27, 1.44]	0.27
**Deprivation**	**[1]OR [95% [2]CI]**	**P-value**
High Deprivation (Ref.)	-	-
Low Deprivation	0.76 [0.57, 1.03]	0.08

. *[1]Odds ratio, [2]Confidence Interval.*

Table S5. Multivariate logistic regression looking at the relationships between ethnicity, and route to diagnosis, and deprivation.

GP Referral vs Emergency Presentation

Ethnicity	[1]OR [95% [2]CI]	P-value
White (Ref.)	-	-
Asian	Not interpretable	Not interpretable
Black	0.48 [0.19, 1.2]	0.12
Mixed & Other	0.23 [0.02, 2.56]	0.23
Unknown	0.85 [0.19, 3.79]	0.83
**Deprivation**	**[1]OR [95% [2]CI]**	**P-value**
High (1st and 2nd Quintiles) (Ref.)	-	-
Low (4th and 5th Quintiles)	1.20 [0.72, 2.03]	0.48

Inpatient Elective vs Emergency Presentation

Ethnicity	[1]OR [95% [2]CI]	P-value
White (Ref.)	-	-
Asian	Not interpretable	Not interpretable
Black	Not interpretable	Not interpretable
Mixed & Other	Not interpretable	Not interpretable
Unknown	Not interpretable	Not interpretable
**Deprivation**	**[1]OR [95% [2]CI]**	**P-value**
High (1st and 2nd Quintiles) (Ref.)	-	-
Low (4th and 5th Quintiles)	0.65 [0.17, 2.56]	0.54

Other Outpatient vs Emergency Presentation

Ethnicity	[1]OR [95% [2]CI]	P-value
White (Ref.)	-	-
Asian	Not interpretable	Not interpretable
Black	0.67 [0.26, 1.73]	0.4
Mixed & Other	1.05 [0.12, 9.14]	0.96
Unknown	0.63 [0.12, 3.20]	0.58
**Deprivation**	**[1]OR [95% [2]CI]**	**P-value**
High (1st and 2nd Quintiles) (Ref.)	-	-
Low (4th and 5th Quintiles)	0.98 [0.57, 1.69]	0.94

Screening vs Emergency Presentation

Ethnicity	[1]OR [95% [2]CI]	P-value
White (Ref.)	-	-
Asian	Not interpretable	Not interpretable
Black	1.22 [0.23, 6.55]	0.82
Mixed & Other	Not interpretable	Not interpretable
Unknown	Not interpretable	Not interpretable
**Deprivation**	**[1]OR [95% [2]CI]**	**P-value**
High (1st and 2nd Quintiles) (Ref.)	-	-
Low (4th and 5th Quintiles)	1.65 [0.62, 4.38]	0.32

TWW vs Emergency Presentation

Ethnicity	[1]OR [95% [2]CI]	P-value
White (Ref.)	-	-
Asian	Not interpretable	Not interpretable
Black	0.08 [0.01, 0.7]	0.02
Mixed & Other	Not interpretable	Not interpretable
Unknown	0.49 [0.07, 3.55]	0.48
**Deprivation**	**[1]OR [95% [2]CI]**	**P-value**
High (1st and 2nd Quintiles) (Ref.)	-	-

Low (4th and 5th Quintiles)	1.31 [0.72, 2.37]	0.38

[1]*Odds ratio,* [2]*Confidence Interval.*

Table S6. Multivariate logistic regression looking for predictors of anal cancer staging (late vs early stage).

Late vs Early Stage		
**Ethnicity**	**[1]OR [95% [2]CI]**	**P-value**
White (Ref.)	-	-
Asian	Not interpretable	Not interpretable
Black	9.64 [0.74, 126.04]	0.08
Mixed & Other	Not interpretable	Not interpretable
Unknown	Not interpretable	Not interpretable
**Deprivation**	**[1]OR [95% [2]CI]**	**P-value**
High (1st and 2nd Quintiles) (Ref.)	-	-
Low (4th and 5th Quintiles)	1.46 [0.47, 4.56]	0.51
**1st Route to Diagnosis**	**[1]OR [95% [2]CI]**	**P-value**
Emergency Presentation (Ref.)	-	-
GP referral	0.23 [0.02, 2.41]	0.22
Inpatient Elective	Not interpretable	Not interpretable
Other Outpatient	0.15 [0.01, 1.74]	0.13
Screening	0.19 [0.01, 3.31]	0.25
2WW	0.14 [0.01, 2.69]	0.19
**Age**	**[1]OR [95% [2]CI]**	**P-value**
<55 years (Ref.)	-	-
>55 years	2.37 [0.63, 8.91]	0.2
**Number of diagnoses**	**[1]OR [95% [2]CI]**	**P-value**
2-3(Ref.)	-	-
4-5	Not interpretable	Not interpretable
**Anal Route to Diagnosis**	**[1]OR [95% [2]CI]**	**P-value**
Emergency Presentation (Ref.)	-	-
GP referral	0.2 [0.02, 1.73]	0.14
Inpatient Elective	Not interpretable	Not interpretable
Other Outpatient	0.15 [0.02, 1.51]	0.11
Screening	Not interpretable	Not interpretable
2WW	1.34 [0.13, 13.79]	0.81
**1st CCI**	**[1]OR [95% [2]CI]**	**P-value**
0 (Ref.)	-	-
1	4.62 [0.6, 35.38]	0.14
2	7.76 [0.56, 106.57]	0.13
3	Not interpretable	Not interpretable
4	Not interpretable	Not interpretable
5	Not interpretable	Not interpretable
**1st Diagnosis**	**[1]OR [95% [2]CI]**	**P-value**
Cervical HSIL (Ref.)	-	-
Anal Cancer	0.68 [0.09, 5.30]	0.71
Anal HSIL	Not interpretable	Not interpretable
Cervical Cancer	0.73 [0.05, 10.75]	0.82
Vaginal Cancer	0.48 [0.00, 52.84]	0.76
Vaginal HSIL	Not interpretable	Not interpretable
Vulval Cancer	0.11 [0.02, 0.68]	0.02
Vulval HSIL	0.21 [0.05, 1.00]	0.05

[1]*Odds ratio,* [2]*Confidence Interval.*

Table S7. Multivariate logistic regression looking for predictors of anal cancer staging (HSIL vs early stage).

	**HSIL vs Early Stage**	
**Ethnicity**	**[1]OR [95% [2]CI]**	**P-value**
White (Ref.)	-	-
Asian	Not interpretable	Not interpretable
Black	1.61 [0.18, 14.55]	0.67
Mixed & Other	Not interpretable	Not interpretable
Unknown	Not interpretable	Not interpretable
**Deprivation**	**[1]OR [95% [2]CI]**	**P-value**
High (1st and 2nd Quintiles) (Ref.)	-	-
Low (4th and 5th Quintiles)	0.85 [0.39, 1.87]	0.69
**1st Route to Diagnosis**	**[1]OR [95% [2]CI]**	**P-value**
Emergency Presentation (Ref.)	-	-
GP referral	3.66 [0.55, 24.36]	0.18
Inpatient elective	0.41 [0.01, 11.62]	0.6
Other Outpatient	2.40 [0.36, 16.24]	0.37
Screening	2.49 [0.24, 25.83]	0.44
2WW	8.66 [0.69, 107.89]	0.09
**Age**	**[1]OR [95% [2]CI]**	**P-value**
<55 years (Ref.)	-	-
>55 years	0.20 [0.08, 0.55]	<0.001
**Number of diagnoses**	**[1]OR [95% [2]CI]**	**P-value**
2-3(Ref.)	-	-
4-5	2.98 [0.28, 31.89]	0.37
**Anal Route to Diagnosis**	**[1]OR [95% [2]CI]**	**P-value**
Emergency Presentation (Ref.)	-	-
GP referral	2.03 [0.26, 15.69]	0.5
Inpatient elective	Not interpretable	Not interpretable
Other Outpatient	3.93 [0.49, 31.78]	0.2
Screening	Not interpretable	Not interpretable
2WW	0.12 [0.01, 1.57]	0.11
**1st CCI**	**[1]OR [95% [2]CI]**	**P-value**
0 (Ref.)	-	-
1	4.15 [0.77, 22.53]	0.1
2	3.37 [0.3, 37.9]	0.32
3	Not interpretable	Not interpretable
4	Not interpretable	Not interpretable
5	0.64 [0.03, 11.6]	0.76
**1st Diagnosis**	**[1]OR [95% [2]CI]**	**P-value**
Cervical HSIL (Ref.)	-	-
Anal Cancer	Not interpretable	Not interpretable
Anal HSIL	Not interpretable	Not interpretable
Cervical Cancer	0.45 [0.05, 4.30]	0.49
Vaginal Cancer	Not interpretable	Not interpretable
Vaginal HSIL	Not interpretable	Not interpretable
Vulval Cancer	1.40 [0.40, 4.96]	0.60
Vulval HSIL	3.17 [1.09, 9.25]	0.03

[1]Odds ratio, [2]Confidence Interval.

Table S8. Multivariate logistic regression looking for predictors of number of anogenital diagnoses.

2-3 vs 4-5 diagnoses		
**Ethnicity**	**[1]OR [95% [2]CI]**	**P-value**
White (Ref.)	-	-
Asian	Not interpretable	Not interpretable
Black	2.17 [0.2, 23.64]	0.52
Mixed & Other	Not interpretable	Not interpretable
Unknown	Not interpretable	Not interpretable
**Deprivation**	**[1]OR [95% [2]CI]**	**P-value**
High (1st and 2nd Quintiles) (Ref.)	-	-
Low (4th and 5th Quintiles)	0.8 [0.14, 4.48]	0.8
**1st Route to Diagnosis**	**[1]OR [95% [2]CI]**	**P-value**
Emergency Presentation (Ref.)	-	-
GP referral	Not interpretable	Not interpretable
Inpatient elective	Not interpretable	Not interpretable
Other Outpatient	Not interpretable	Not interpretable
Screening	Not interpretable	Not interpretable
2WW	Not interpretable	Not interpretable
**Age**	**[1]OR [95% [2]CI]**	**P-value**
<55 years (Ref.)	-	-
>55 years	1.74 [0.14, 21.07]	0.66
**1st Diagnosis**	**[1]OR [95% [2]CI]**	**P-value**
Cervical HSIL (Ref.)	-	-
Anal Cancer	Not interpretable	Not interpretable
Anal HSIL	Not interpretable	Not interpretable
Cervical Cancer	Not interpretable	Not interpretable
Vaginal Cancer	Not interpretable	Not interpretable
Vaginal HSIL	0.92 [0.03, 25.27]	0.96
Vulval Cancer	Not interpretable	Not interpretable
Vulval HSIL	0.13 [0.02, 0.91]	0.04
**1st CCI**	**[1]OR [95% [2]CI]**	**P-value**
0 (Ref.)	-	-
1	Not interpretable	Not interpretable
2	Not interpretable	Not interpretable
3	Not interpretable	Not interpretable
4	12.44 [0.44, 353.06]	0.14
5	Not interpretable	Not interpretable
**1st Treatment**	**[1]OR [95% [2]CI]**	**P-value**
CRT (Ref.)	-	-
Cryotherapy	Not interpretable	Not interpretable
Laser	Not interpretable	Not interpretable
Radiotherapy	Not interpretable	Not interpretable
Surgery	Not interpretable	Not interpretable

[1]*Odds ratio*, [2]*Confidence Interval*
